# Pregnancy and antibody-mediated CNS disorders: What do we know and what should we know?

**DOI:** 10.3389/fneur.2022.1048502

**Published:** 2022-12-19

**Authors:** Rosa Cortese, Sara Mariotto, Chiara Rosa Mancinelli, Carla Tortorella

**Affiliations:** ^1^Department of Medicine, Surgery and Neuroscience, University of Siena, Siena, Italy; ^2^Neurology Unit, Department of Neuroscience, Biomedicine and Movement Sciences, University of Verona, Verona, Italy; ^3^Multiple Sclerosis Center, Spedali Civili of Brescia, Brescia, Italy; ^4^Department of Neurosciences, S. Camillo-Forlanini Hospital, Rome, Italy

**Keywords:** neuromyelitis optica spectrum disorders (not in MeSH), autoimmune encephalitis, myelin oligodendrocyte (MOG) antibody associated disease, pregnancy, postpartum, breastfeeding

## Abstract

Antibody-mediated central nervous system (CNS) disorders including those associated with aquaporin-4 or myelin oligodendrocyte glycoprotein IgG and autoimmune encephalitis often affect women of childbearing age. Pathogenic antibodies of these diseases can potentially alter reproductive functions and influence fetal development. Hormonal changes occurring during pregnancy may modify the course of autoimmune diseases by influencing relapse risk, attack severity, and affect the delivery and postpartum period. Moreover, balancing treatment related safety issues with the risk of potentially disabling relapses during pregnancy and breastfeeding are major challenges. Intentional prenatal, gestational, and post-partum counseling is paramount to address these issues and mitigate these risks. Fortunately, new insights on risk factors for adverse pregnancy outcomes and possible preventive strategies are emerging. This review aims to summarize the interplay between antibody-mediated CNS disorders and pregnancy during the prenatal, gestational, and postpartum periods, highlight current treatment recommendations, and discuss future areas of research.

## Introduction

The most common Antibody (Ab)-mediated disorders of the central nervous system (CNS) are aquaporin-4 antibody neuromyelitis optica spectrum disorders (AQP4+NMOSD), myelin-oligodendrocyte glycoprotein antibody-associated disease (MOGAD), and autoimmune encephalitis (AE). These diseases are often diagnosed in women of childbearing age (1–3).

Pregnancy-related hormonal fluctuations can influence autoimmune diseases by altering the disease course, while pregnancy itself can significantly impact treatment options in clinical practice. Conversely, pathogenic antibodies of Ab-mediated diseases may alter fertility and reproductive function as well as target the placenta, contributing to adverse pregnancy outcomes and altering fetal development. These factors should be carefully evaluated in women with Ab-mediated disorders to provide comprehensive pre-pregnancy planning, minimize disease activity, and disease burden during gestation, and optimize successful delivery and child and maternal health in the post-partum period.

**Figure 1:**
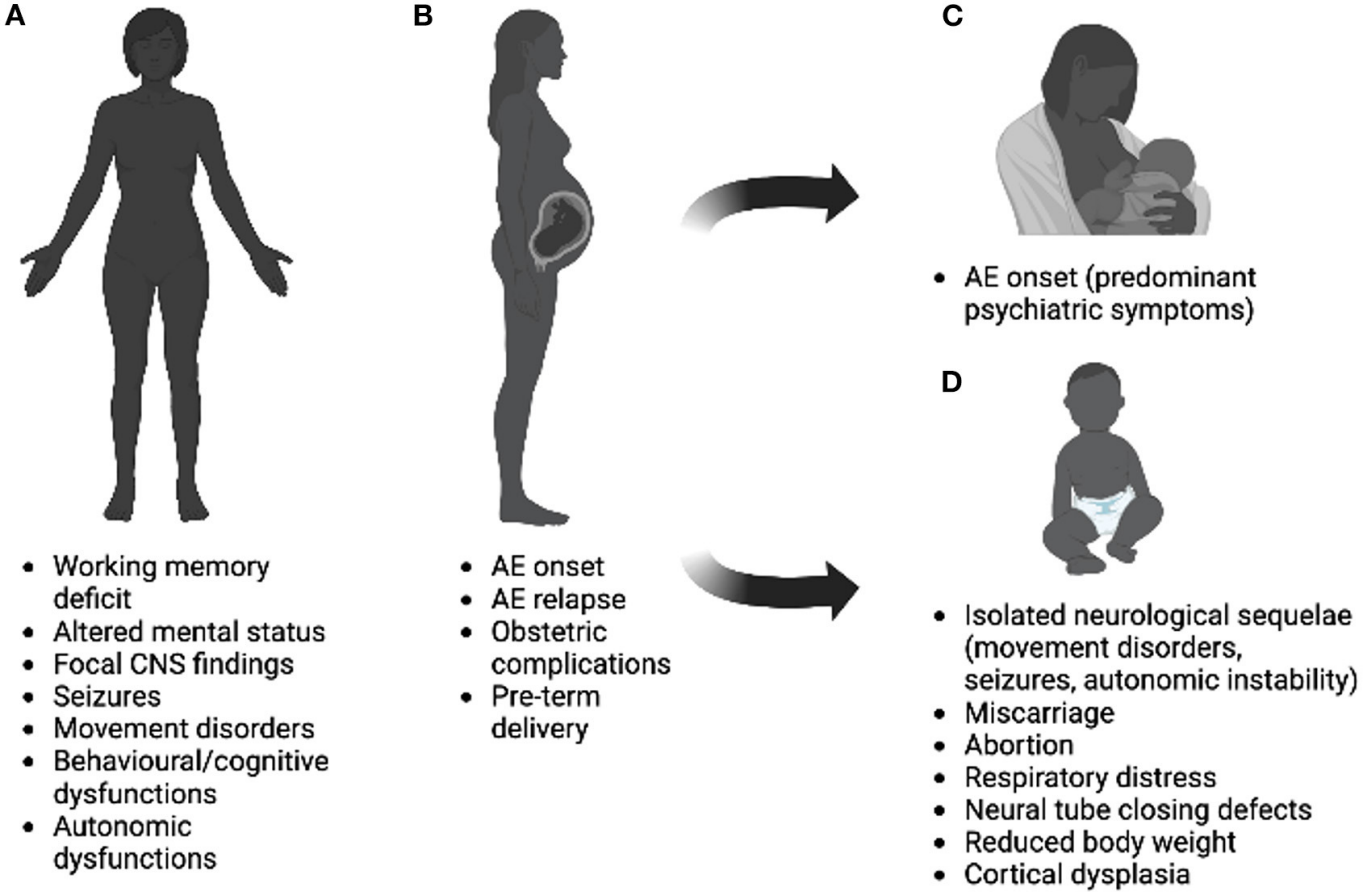
Interplay between pregnancy and autoimmune encephalitis on women before **(A)**, during **(B)**, after **(C)** pregnancy, and effects on newborns **(D)**. Possible effects illustrated in the figure refer to rare cases, mostly linked to anti-NMDAR encephalitis and can results from the cumulative effect of antibodies, sedatives, and antiepileptic drugs. Image was created using Biorender.com.

The aim of this review is to summarize the interplay between Ab-mediated diseases and expectant mothers during the prenatal, gestational, and postpartum periods, highlight current treatment recommendations, and discuss emerging insights and research. As the interactions between the underlying disease and pregnancy can vary between these conditions, we will summarize the key elements separately for AQP4+NMOSD, MOGAD, and AE. For each disease, we will discuss (i) the current state of the art (i.e., “what do we know”) regarding fertility, pregnancy, the postpartum period and treatment strategies, and (ii) future areas of research (i.e., “what should we know”). Table 1 summarizes currently available studies reporting data on pregnancy-related outcomes in AQP4-Ab- and MOG-Ab-mediated disorders; Table 2 provides an overview on the impact of the diseases on pregnancy and vice versa. Figure 1 summarizes the interplay between pregnancy and AE. Box 1 highlights future areas of research.

**Table 1:** Currently available studies assessing the relationship between aquaporin 4 (AQP4) and myelin-oligodendrocyte glycoprotein (MOG) antibody-mediated disorders of the central nervous system (CNS) and pregnancy.

**References**	Number^*^of patients included (AQP4+/MOG+/ seronegative)	**Number of pregnancies**	**Design of the study**	**Countries involved**	**Outcomes**
Kim et al. (4)	40 (40/0/0)	54	Retrospective multicenter international study	Korea, Japan, United Kingdom, Portugal	•Mean ARR before, during and after (up to 6 months) pregnancy •Maternal outcomes
Bourre et al. (5)	20 (8/0/12)	25	Retrospective multicenter national study	France	•Mean ARR before, during and after (up to 1 year) pregnancy •EDSS before and after (up to 1 year) pregnancy •Effect of epidural analgesia and breastfeeding on disease activity
Fragoso et al. (6)	17 (NA)	17	Retrospective multicenter national study	Brazil	•Relapse rate before, during and after (up to 1 year) •EDSS before and after (up to 1 year) pregnancy •Maternal outcomes
Shimizu et al. (7)	47 (47/0/0)	56	Retrospective multicenter study	Japan	•ARR before, during and after (up to 1 year) pregnancy •Maternal outcomes •AQP4 antibody serostatus in the newborns
Nour et al. (8)	60 (60/0/0)	126	Retrospective multicenter international study	United Kingdom, Portugal, Japan	•Maternal outcomes
Huang et al. (9)	55 (NA)	63	Retrospective multicenter national study	South China	•ARR before, during and after (up to 1 year) pregnancy •EDSS before and after (up to 1 year) pregnancy •Maternal outcomes •AQP4 antibody serostatus in the newborns
Klawiter et al. (10)	31 (25/0/6)	46	Retrospective multicenter national study	United States, United Kingdom, Germany	•ARR before, during and after (up to 9 months) pregnancy •Maternal outcomes
Delgado-García et al. (11)	19 (19/0/0)	50	Retrospective multicenter national study	Mexico	•Maternal outcomes
Tong et al. (12)	128 (NA)	234	Retrospective single center study	China	•Mean ARR before, during and after (up to 1 year) pregnancy •Maternal outcomes
Ashtari et al. (13)	11 (11/0/0)	20	Retrospective single center study	Iran	•Mean ARR before, during and after (up to 1 year) pregnancy •Maternal outcomes
Kim et al. (14)	26 (NA)	33	Retrospective single center study	Korea	•Maternal outcomes •Risk factors predicting pregnancy-related attacks
Wang et al. (15)	110 (83/21/6)	136	Retrospective single center study	China	•Mean ARR before, during and after (up to 1 year) pregnancy •EDSS score before and after (up to 1 year) pregnancy •Risk factors predicting pregnancy-related attacks
Collongues et al. (16)	58 (46/30/13)	89	Retrospective multicenter international study	United Kingdom, France, Portugal	•Mean ARR before, during and after (up to 1 year) pregnancy •Maternal outcomes •Effect of immunosuppressive therapy on relapse risk
Jarius et al. (17)	5 (0/5/0)	7	Retrospective multicenter international study	Germany, Denmark, Italy, Austria	•Number of relapses during and after (up to 6 months) pregnancy

* Numbers include informative patients.

AE, autoimmune encephalitis; AQP4, aquaporin4; ARR, annualized relapse rate; EDSS, expanded disability status scale; MOG, myelin- oligodendrocyte glycoprotein antibody.

**Table 2:** Impact of each Ab-mediated disease on pregnancy and vice versa.

	**Pre-conception**	**Pregnancy**		**Post-partum**		**Comments/limitations**
	**Effect of disease on fertility**	**Effect of disease on pregnancy**	**Effect of pregnancy on disease**	**Effect of disease on post-partum**	**Effect of post-partum on disease**	
**AQP4+NMOSD**	•Reduced fertility because of: - Advanced age at conception - Previous immunosuppressive treatment - AQP4-related hypothalamic dysfunction - High disability with sphincter and sexual dysfunction	•Risk of poor pregnancy outcomes due to: - AQP4-Ab related placentitis - Concomitant autoimmune conditions (e.g., systemic lupus erythematosus/ antiphospholipid syndrome) •Type of delivery influenced by: - High disability	•Contrasting data (reduced risk of relapses compared to the pre-pregnancy period?) •Onset of disease due to: - Changes in hormonal and status - Change in B/T-cells ratios	•Neonatal complications, including low birth weight and stillbirth •AQ*P4*-Ab detected transiently in the blood of new-borns at birth (no associated symptoms described) •Permanent or transitory effect of immunosuppressive treatment during pregnancy on neonatal outcomes	•Onset of disease or increased risk of acute relapse due to: - Changes in hormonal status - Change in B/T-cells ratios •No effect of type of delivery and obstetrical analgesia •No influence of breastfeeding	- Few studies including small number of patients, mainly retrospective, often not corrected for confounding factor
**MOGAD**	•Reduced fertility because of: - Residual symptoms from spinal cord relapse - Previous immunosuppressive treatment	•Risk of poor pregnancy outcomes due to: - Residual sphincter dysfunction with related risk of infection •No effect of type of delivery and obstetrical analgesia	•Contrasting data (reduced risk of relapses compared to the pre-pregnancy period?) •Onset of disease due to: - Changes in hormonal and status - Change in B/T-cells ratios	•No direct data available	•Onset of disease or increased risk of acute relapse due to: - Changes in hormonal status - Change in B/T-cells ratios	•Few studies including small number of patients, mainly retrospective, often not corrected for confounding factor
**Autoimmune encephalitis**	•Reduced fertility because of: - Residual symptoms - Previous immunosuppressive treatment - Socioeconomic factors - Surgical treatment of the tumor in paraneoplastic anti-NMDAR encephalitis associated with ovarian teratoma	•Risk of poor pregnancy outcomes due to: - Severity of the mother disease - Maternofetal transfer of anti-NMDAR antibodies	•Contrasting data (reduced risk of relapses compared to the pre-pregnancy period?) •Onset of disease or increased risk of acute relapse due to: - Changes in hormonal status - Different B/T cells balance and cytokine production - Modification of immune tolerance	•Neurological sequel Miscarriage due to: - Severity of the mother disease - Treatment administered to the mother (e.g., antiepileptic and sedative) - transfer of anti-NMDAR antibodies	•Onset of disease or increased risk of acute relapse due to: - Changes in hormonal status - Different B/T cells balance and cytokine production - Modification of immune tolerance	•Few reported cases, most of patients with anti-NMDAR encephalitis

Box 1 **Summary of areas for future research in the three diseases.**

All the Ab-mediated diseases included in this review are rare disorders. Multicenter and possibly registry-based real-word studies are needed to assess the impact of pregnancy on CNS autoimmune mediated disorders and vice versa

Future works should:

- Stratify NMOSD patients by serotype (AQP4-Ab seropositive, seronegative, and double seronegative)
- Evaluate MOGAD separately, as this is now recognized as a distinct disease
- Evaluate if clinical features of NMOSD, MOGAD, or AE (i.e., different phenotypes at onset, number and recovery of relapses, age at conception, treatment) or Ab-characteristics (titers, timing, and persistence over time) may be independent risk factors for pregnancy-related relapses
- Clarify the correlation between mother's serum antibody titer and obstetric or fetal complications to facilitate pre-conceptual planning and postpartum follow-up
- Define guidelines for the treatment of NMOSD, MOGAD, and AE before, during and after pregnancy
- Provide a detailed long-term neuropsychological assessment of children in all Ab-mediated diseases of the CNS to define short and long-term outcomes.

Content for this paper was gathered by PubMed literature review for articles published between 2012 and 2022 with the following search terms: “aquaporin 4,” “neuromyelitis optica spectrum disorder,” “myelin- oligodendrocyte glycoprotein antibody-associated disease,” “autoimmune encephalitis,” “NMDAr,” “LGI1,” “CASPR2,” “fertility,” “partum,” “delivery,” “postpartum,” “pregnancy,” “breastfeeding,” “fetus,” “gestation,” and “treatment.”

## Aquaporin-4 antibody neuromyelitis optica spectrum disorders

Neuromyelitis optica (NMO; previously known as Devic's disease) is a severely disabling Ab-mediated astrocytopathy with secondary demyelination (18, 19). Anti-AQP4 antibodies (AQP4-Ab) have been recognized as the pathogenic hallmark of the disease. The term of NMO spectrum disorders (NMOSD) has been recently adopted to identify conditions in which CNS involvement is not restricted to the optic nerves and spinal cord (1).

The currently established international criteria for NMOSD allow for a minority of patients with no detectable anti-AQP4-Ab to be diagnosed as seronegative NMOSD if specific clinical and magnetic resonance imaging (MRI) requirement are satisfied and alternative diagnoses have been excluded (1). Pathophysiological mechanisms underlying seronegative cases remain unclear, but likely differ from those of seropositive NMOSD (20, 21). Studies report that 10–40% of seronegative NMOSD patients have IgG Ab against myelin oligodendrocyte glycoprotein (MOG) and distinct clinical, epidemiologic and radiological features (17, 22).

NMOSD associated with AQP4-Ab (AQP4+NMOSD) is a rare condition with a prominent female predominance (female-to-male ratio: from 9:1 to 10:1) (20, 23). Genetic, epigenetic or hormonal factors can contribute to female susceptibility as in other autoimmune disorders (24, 25). The typical onset of AQP4+NMOSD occurs between the third and fourth decades of life, when many women are still considering childbearing (23, 26) and highlights the importance of understanding the effects of this disease on fertility, pregnancy, and the postpartum period.

### What do we know?

#### Effect of Ab-mediated disease on fertility and pregnancy

The impact of AQP4+NMOSD on fertility is unclear and poorly investigated. In a multicenter study, Bove et al. evaluated 217 NMOSD women (82% AQP4-Ab seropositive) using a standardized reproductive survey. Six percentage of these patients received infertility treatment, while 13% reported a delay of at least 12 months in achieving pregnancy (27). However, the mean age of NMOSD patients at onset was 40 years and the age at conception was not specified, thus biasing the results for age related infertility which is known to rise with increasing female age (28). Another study measured anti-Mhormone as a marker of ovarian reserve and found that levels were reduced in 14 NMOSD patients (11 AQP4-Ab seropositive, 2 AQP4-Ab seronegative, 1 MOG-Ab seropositive) when compared to 8 healthy controls. However, data were not corrected for factors potentially influencing fertility such as comorbidities or immunotherapies, which limits the generalizability of these findings (29).

AQP4 has variable expression in the female reproductive tracts of adult mammals, having been identified in the uterus and cervix, but not in the ovaries (30). Although AQP4 is thought to be involved in the physiology and pathophysiology of the reproductive system and in pregnancy (30), the exact mechanisms by which AQP4-Ab can affect female fertility remains unknown. In an experimental study, female AQP4-knockout mice showed reduced fertility with defective folliculogenesis, reduced corpora lutea formation, and decreased uterine response to gonadotropins, probably related to a dysregulation of the hypothalamus-pituitary-ovary axis (31). This may be explained by the fact that AQP4 is highly expressed in some brain regions, such as the periventricular area and the paraventricular hypothalamic nucleus, which are involved in the regulation of gonadotropin-releasing hormone neurons and influence the secretion of sexual hormones (32). Alternatively, hypothalamic dysfunctions and secondary endocrinopathies influencing fertility might affect NMOSD patients with diencephalic lesions (33). Finally, some immunosuppressive drugs such as cyclophosphamide or mitoxantrone may be linked to changes in female fertility and ovarian reserve.

Another fertility consideration is the possible effect of AQP4 expression on the placenta and transplacental migration. Autoantibodies, such as the acetylcholine receptor-Ab or Anti-Ro-Ab, can be transmitted to the fetus through transplacental transportation, leading to pregnancy complications and influencing perinatal outcomes. Whether placental or fetal damage may be due to AQP4-Ab exposure is not known. Retrospective studies, including mainly AQP4-Ab cases, showed that NMOSD patients have an increased rate of pregnancy complications, including the risk of miscarriage (8, 10, 11). In particular, one large retrospective study of 60 AQP4+NMOSD women with 126 pregnancies found that pregnancies after NMOSD onset were associated with an increased risk of miscarriage when compared to pregnancies before the onset, independently from the risk associated with an advanced maternal age, particularly in those patients with high disease activity before conception and during pregnancy (8). The exact mechanism underlying miscarriage in NMOSD is also unknown. AQP4-Ab could be a possible causative agent of spontaneous abortion, as AQP4 is expressed in the syncytiotrophoblast of human and mouse placenta, especially during the second trimester. In animal models, the transfer of human AQP4-IgG bound mouse placental AQP4, activated coinjected human complement and led to the induction of placentitis and fetal death (34). In parallel, several regions of necrosis with multiple infarcts were observed in a patient with NMOSD who miscarried in the second trimester, mainly in the maternal part of the placenta (35). AQP4 immunostaining showed a complete loss of immunoreactivity. High titers of circulating antibodies in the context of active disease could favor placental damage and miscarriage. Contrasting results are reported by another recent study including six pregnant AQP4+NMOSD patients, their infants, and three healthy controls. Histological investigation showed no significant difference in the intensity of the immunohistochemical staining for AQP4, and in inflammatory markers in placentae of patients and controls. Four of the six patients were term pregnancy and their infants had a normal development despite showing AQP4-Ab at the time of birth (36). Taking these preliminary data into account, the hypothesis suggesting that AQP4-Ab may cause placentitis with a risk of miscarriage should be further investigated.

The rate of preeclampsia in NMOSD was found to be similar to that of the general population (16), whereas its risk increased in women with at least two other concomitant autoimmune diseases, regardless of NMOSD onset. Additional autoimmune conditions are reported in 20–30% of patients with NMOSD (37), some of which (e.g., systemic lupus erythematosus and antiphospholipid syndrome), are known risk factors for preeclampsia in the general population (38).

AQP4-Ab crosses the placental barrier and can be detected in the blood of newborns at birth. However, infants become seronegative 1–3 months later and usually do not have neurological symptoms (7, 39, 40).

The available evidence on fetal outcomes in NMOSD patients is scarce. In a systematic review and meta-analysis neonatal complications, including low birth weight and stillbirth, were described in 33/619 (5.3%) of the informative pregnancies (41). Among these, immunosuppressive treatment during pregnancy was associated with neonatal complications in 13 events. The possible teratogenic effects of some drugs used in NMOSD during pregnancy need to be specifically evaluated (42).

#### Effect of pregnancy, postpartum, breastfeeding on Ab-mediated disease

There is a growing number of retrospective studies evaluating the effect of pregnancy and the postpartum period on NMOSD. While they all report an increased relapse rate postpartum, data on the interplay between the gestation period and the disease are not conclusive (4–7, 9, 10, 12, 15). Differences between study findings may be due to the heterogeneity of the cohorts, including seronegative NMOSD patients as well as those with different treatment history and onset during gestation.

Recently, a retrospective multicenter study assessed the effect of pregnancy on 58 women with NMOSD, stratifying patients by antibody status (AQP4-Ab, MOG-Ab, double-seronegative). Eighty-nine pregnancies were observed. Patients had a reduced risk of relapse during pregnancy in each serostatus group when compared to the pre-pregnancy period, while the annualized relapse rate was higher during the first postpartum trimester only in AQP4-Ab positive women (16). Factors associated with a reduced risk of NMOSD attacks were: being on an immunosuppressive treatment during pregnancy and an older age at conception (7, 41, 43, 44).

The mechanisms underlying the effect of pregnancy on NMOSD have not been sufficiently investigated. It has been suggested that high estrogen levels may influence AQP4-Ab type, titer, and glycosylation pattern, as well as stimulate the differentiation of antibody-producing B-cells. Moreover, a shift toward a Th2-mediated immunity, which occurs during pregnancy, could sustain NMOSD pathogenesis (45).

To date, there are no studies specifically investigating the effect of obstetrical analgesia or type of delivery on disease activity in NMOSD. However, retrospective and observational data suggest that cesarean delivery, spinal, or epidural anesthesia do not affect the disease (4, 5, 9, 10). Likewise, breastfeeding does not appear to influence on the disease course (4, 5). Given the paucity of data, the management of patients with NMOSD could be guided by extensive experience in multiple sclerosis (MS). In MS, the type of delivery and anesthetic options are not influenced by the disease unless there is significant disability and is based on obstetric criteria (46).

In conclusion, current evidence indicates that pregnancy is associated with a risk of relapse in women with AQP4-Ab NMOSD, especially in the postpartum period and in young women with no previous immunosuppressive treatment. Due to the rarity of the disease, prospective and large cohort studies are scarce.

#### Treatment strategy change during pregnancy and impact on Ab-mediated disease

Relapses can be devastating in NMOSD resulting in permanent disability. Thus, prevention and timely treatment of relapses is of primary importance. During pregnancy and breastfeeding, specific considerations and a careful risk-benefit analysis should be performed, considering the possible effects of drugs on the fetus and infant. To date, specific treatment guidelines are not available. However, a detailed review with recommendations based on available evidence and expert opinion on the therapeutic management of NMOSD during pregnancy have recently been published (42).

Acute treatment of relapses with methylprednisolone, plasma exchange and immunoadsorption is possible during pregnancy and breastfeeding. Corticosteroids may cross into the fetal circulation, depending on the type of steroids administered, with low risk when non-fluorinated corticosteroids such as prednisone, prednisolone, and methylprednisolone are used after the first trimester. Since corticosteroid levels in the breast milk are low, lactation is also feasible. Nevertheless, it would be advisable to delay breastfeeding for at least 4 h from treatment if high doses of methylprednisolone are administered (42).

Plasma exchange has been used in pregnant women with other Ab-mediated conditions, such as antiphospholipid Ab-syndrome and thrombotic thrombocytopenic purpura, with no evidence of increased risk of adverse effects. This treatment is considered relatively safe for acute NMOSD relapse, but an accurate risk-benefit evaluation is advised, with the same indications applied for immunoadsorption (42).

Considering the effect of pregnancy on the course of NMOSD, especially in the postpartum period, the immunosuppressive treatment choice before conception and the decision to stop or continue these drugs during pregnancy and after delivery requires careful evaluation. Immunosuppressant treatment discontinuation or insufficient immunosuppression have been proposed as risk factors for NMOSD attacks during or soon after pregnancy (7, 41, 44). Yet, NMOSD treatments may be harmful to the fetus and many lack adequate safety data in this population.

Cyclophosphamide and mitoxantrone are contraindicated during pregnancy and breastfeeding. Due to their potential ovarian toxicity, they should also be avoided in women of childbearing age.

Mycophenolate mofetil and methotrexate carry a high risk of miscarriage and congenital malformations. If necessary, an appropriate wash-out period before attempted conception must be ensured, while breastfeeding should be avoided in women on these treatments (42, 47).

Azathioprine has been deemed a relatively safe therapy during pregnancy and lactation based on a large number of exposed pregnant women with several other autoimmune conditions. After a risk-benefit evaluation, especially in active NMOSD patients, continuing azathioprine may be considered (42, 47).

Rituximab readily crosses the placenta from the second trimester and depletes fetal B-cells, an effect that reverts within 6 months from birth (48). Recently, it has been demonstrated that rituximab, administered within 6 months of conception or during pregnancy in more than 100 women with MS or NMOSD, was not associated with an increased risk of adverse outcomes (49). Considering the growing evidence on the use of anti-CD20 therapies in women of childbearing age, the use of rituximab in selected cases might be considered, ensuring a period of at least 3 months between the last infusion and conception (49, 50), or less as suggested by some experts (42). Moreover, IgG1-based monoclonal antibodies are minimally transferred into breastmilk. In particular, the “relative infant dose” for rituximab is < 0.4% and significantly less than the acceptable threshold of 10%, alluding to its probably safety during breastfeeding (51).

Data on the safety of tocilizumab use during pregnancy come primarily from patients with rheumatological diseases. In an analysis from a global safety database, prospective, and retrospective data on pregnancies during tocilizumab treatment showed a slightly increased risk of miscarriage, preterm birth, and malformations without a distinct pattern. However, one third of these patients with adverse outcomes were concomitantly treated with methotrexate and leflunomide (52). Of the few reports concerning breastfeeding in tocilizumab treated patients, none have reported negative effects on the infant. Therefore, the American College of Rheumatology concluded that treatment until conception and breastfeeding during treatment are supported by conditional evidence (53). To date, a single report described a double-seronegative NMOSD woman with a highly active disease course who continued tocilizumab until the 28th week of gestation and resumed infusions 4 days after delivery. Her pregnancy course was clinically unremarkable and no congenital malformation nor hematological alterations were detected in the infant up to 1 year of age (54). Based on this data, NMOSD experts suggest tocilizumab can be used during pregnancy in patients with severe disease and during breastfeeding, after a careful risk-benefit analysis has been conducted.

To date, there is scant data concerning pregnancy and breastfeeding in women with NMOSD treated with new monoclonal antibodies (mAbs). However, clues from other indications and drugs with similar mechanisms of action are available. Satralizumab, a IL-6 receptor mAb, is expected to have no specific teratogenic effects in humans, due to its similar mechanism of action with tocilizumab. Similarly, inebilizumab, an anti-CD19 mAb, may have a comparable safety profile in pregnancy and on the newborn to other B-cell-depleting. Data from a ten-year real world hematological registry of eculizumab, a mAbs against the C5 fraction of complement, suggest that the rate of live births without fetal/maternal complications associated with eculizumab-exposure during pregnancy and the postpartum period were consistent with that of the general population (55). Moreover, eculizumab is not detected in breast milk samples (56), making it a potential option in pregnant and breastfeeding women with NMOSD.

To summarize, severe relapses may be treated during pregnancy in NMOSD, and due to the high risk of relapses in the postpartum period, when the immunosuppressant treatment has been stopped, an early resumption of the drug should be carefully evaluated.

### What should we know

#### Future areas of research

Despite the growing interest on the effect of pregnancy on NMOSD and *vice versa*, available studies are mainly retrospective and include a relatively small number of cases. Given the rarity of this condition, international and collaborative prospective registries are needed. Moreover, rigorous inclusion criteria should be shared among studies to include more homogenous groups. Separating AQP4 seropositive from seronegative NMOSD patients would also be advantageous acknowledging the limited sample sizes of this approach. In the future, pregnant women should be further stratified according to their ethnicity and clinical factors (i.e., number and severity of previous attacks, use of immunosuppressive treatments) to assess whether these factors may influence the disease course.

Immunological changes occurring during pregnancy and the role of pregnancy-related hormones should be investigated to better understand their possible effect on NMOSD immunopathogenesis. For example, any variation in immune regulatory cells and complement, as well as in IL-6 and neurofilament light chain levels, evaluated during pregnancy may be informative. Speculations on this topic have all focused on AQP4 autoimmunity. The role of the placenta as a possible source of AQP4 in disease onset and in triggering relapses should be elucidated, as well as the relationship between the transfer of AQP4-Ab into the placenta and complications of pregnancy.

Finally, the decision to stop or continue immunosuppressive treatment during pregnancy and breastfeeding remains controversial. Considering the efficacy and the effect on specific targets of immunopathogenesis of AQP4+NMOSD, guidelines on the use of the new approved mAbs during pregnancy are necessary.

## Myelin-oligodendrocyte glycoprotein antibody-associated disease

Myelin-oligodendrocyte glycoprotein antibody-associated disease (MOGAD) is a distinct autoimmune demyelinating disorder of the CNS characterized by the presence of a pathogenic autoantibody against a CNS-specific protein located in the outer layers of the myelin sheath (2). The immune attack of MOG-Ab is associated with myelin and oligodendrocyte damage, leading to wide and heterogeneous clinical manifestations in both pediatric and adult patients (22, 57). MOGAD can be either monophasic or relapsing, and its typical clinical phenotypes include acute disseminated encephalomyelitis (ADEM), isolated transverse myelitis, isolated optic neuritis (ON), and unilateral cerebral cortical encephalitis with epilepsy (22, 58, 59). Due to its rarity and relatively recent identification as a distinct disease, our knowledge on the epidemiology of MOGAD is still evolving. In general, MOGAD occurs in greater frequency in younger people compared with AQP4+NMOSD (60). Unlike AQP4+NMOSD, which has a significant female predominance, most studies have shown that MOGAD equally affects males and females in young children (age < 10 years), with a slight female predominance in older post-pubertal children and adults (61). MOGAD patients have been identified in the cohorts of seronegative NMOSD in previous works, which did not separately analyze results by antibody type. Therefore, studies assessing the effect of MOG-Ab on pregnancy and of pregnancy on MOGAD disease course are both limited and conflicting.

### What do we know

#### Effect of Ab-mediated disease on fertility and pregnancy

To date, there is almost no data concerning the effect of MOG-Ab on fertility and pregnancy. As for AQP4, MOG antigens can be also found in the placenta, and MOG-Ab crosses the placental barriers during the second and third trimesters. MOG-Ab are likely to be transferred to the fetus and found in the blood of the newborn (42). However, their role in the pathophysiology of pregnancy is still unknown.

MOG-specific B-cells in the peripheral immune system produce MOG-Ab, which cross the blood-brain barrier (BBB) and enter the CNS where they bind MOG on oligodendrocytes, leading to myelin injury and demyelination. T-cells are also involved in the pathogenesis of MOGAD. Indeed, T helper cells are needed for the differentiation of B-cells into specific plasma cells, as human MOG-IgG are mainly of the IgG1 phenotype. Moreover, MOG-specific CD4+ T-cells or myelin basic protein-specific T effector cells and macrophages in the CNS are increasingly activated and cytokines and chemokines levels enhanced further propagating the immune reaction. During pregnancy, hormonal fluctuations can change B- and T-cells ratios potentially inducing inflammation and increasing pregnancy complications (42). The role of hormonal-related changes in pregnant MOGAD patients requires further evaluation.

In MOGAD, disease phenotype, relapse risk, relapse severity, and degree of recovery are all age dependent. Children more frequently experience brain involvement, a monophasic disease course, worse severity, and faster recovery than adults. In women of childbearing age, relapses preferentially involve the optic nerve and spinal cord, particularly the lower cord, and conus medullaris (2, 62). In a retrospective study assessing the clinical outcomes of transverse myelitis, Mariano et al. showed that the overall mobility recovery was better in patients with MOGAD than AQP4+NMOSD, but sphincter dysfunction remained a significant characteristic in MOGAD (63). This residual dysfunction may increase the risk of infections, with potential complications in pregnancy.

MOGAD patients are rarely treated with aggressive immunosuppressive treatments, such as cyclophosphamide, which may reduce ovarian reserve and fertility. However, this should be taken into consideration in clinical practice, and when immunosuppressants are needed, alternative therapeutic options should be preferred in young women patients.

In summary, the effect of MOG-Ab on fertility, pregnancy, and the newborn need to be further elucidated. In MOGAD women, disease characteristics as well as residual symptoms from previous cord relapses may complicate pregnancy.

#### Effect of pregnancy, postpartum, breastfeeding on Ab-mediated disease

Currently, there are only a few reports and two systematic studies on the effect of pregnancy and the postpartum period on MOGAD.

An association between assisted reproductive technology with frozen embryo transfer (FET) and the first manifestation of MOG optic neuritis in a previously healthy patient with unexplained infertility was recently proposed (64). This patient experienced bilateral optic neuritis after a single FET, which recovered completely after intravenous steroids and plasma exchange. However, further studies are needed to confirm this potential association.

A retrospective multicenter study of 30 patients with MOGAD reported one or more relapses in about half of MOGAD women with a documented pregnancy (5/10) most of them in the postpartum period within 8 months after delivery. Interestingly, while the cases that occurred during pregnancy were in patients with an already diagnosed relapsing MOGAD disease course, the disease started postpartum in three other patients (17). This may suggest that immunological changes related to pregnancy and delivery may play a role in triggering relapses and inducing the disease.

Recently, the effect of pregnancy on MOGAD was systematically evaluated in two independent cohorts. One study involving a Caucasian cohort from France, the United Kingdom, and Portugal, found that the annualized relapse rate (ARR) was lower during pregnancy than pre-pregnancy in all NMOSD serostatus groups, including 30 MOGAD patients, but rebounded during the first postpartum trimester. Unlike AQP4+NMOSD, immunosuppressant treatment during pregnancy or postpartum did not reduce the risk of relapses in MOGAD, though the majority of patients in this cohort were untreated (16).

Similarly, a Chinese study including 21 patients with MOGAD, showed that the first postpartum trimester was the highest risk period for relapse, and that the relapse risk during the first year postpartum was 1.5 time higher compared to 1 year pre-pregnancy. The pregnancy-related relapses in this MOGAD cohort were characterized by more episodes of optic neuritis, but fewer episodes of acute myelitis than the AQP4+NMOSD cohort. While the disability level during the pregnancy-related relapses did not differ between the two disease cohorts, EDSS scores were lower in the remission phase in MOGAD patients, suggesting a better recovery than AQP4+NMOSD even during pregnancy. In this study, only one premature delivery was observed with no spontaneous abortion, neonatal malformations, or pre-eclampsia reported (15).

Recently, an isolated case of MOGAD presenting 3 weeks postpartum with bilateral optic neuritis and a history of SARS-COVID19 infection 1 week before the delivery has been described (65). In another recently reported case, MOG-Abs were found in a patient with systemic lupus erythematosus and thoracic longitudinally extensive transverse myelitis 1 month postpartum. This case illustrates that the two diseases can coexist and the postpartum state may have facilitated the onset of the both autoimmune conditions (66).

A severe postpartum rhombencephalitis presenting 6 months after delivery in an undiagnosed patient with a history of recurrent LETM was found to be MOG-Ab positive. Symptoms improved after plasmapheresis with complete resolution of the infratentorial lesion and no relapses after 1 year on long-term immunosuppression with azathioprine (67). A Japanese patient diagnosed with MOGAD following cortical encephalitis, experienced increased seizure frequency in the 2 months postpartum of two pregnancies, despite treatment with levetiracetam. The umbilical cord blood of the second child was positive for MOG-Ab (68).

To date, little is known about MOGAD and breastfeeding, with no systematic study assessing the relationship between the two conditions.

To summarize, a rebound of disease activity during the first postpartum trimester and potentially up to 6 months postpartum is frequent in MOGAD, but further studies are needed to explore the role of other pregnancy-related factors in this Ab-mediated disorder.

#### Treatment strategy change during pregnancy and impact on Ab-mediated disease

Currently, there are no clinical trials for the treatment of MOGAD. Management strategies are primarily based on repurposing medications from other autoimmune diseases of the CNS.

Like AQP4+NMOSD, acute relapses should be promptly treated in MOGAD with intravenous methylprednisolone followed by escalation therapies, such as plasma exchange in patients with severe attacks or incomplete recovery (2).

Conversely, while all patients with AQP4+NMOSD require long-term immunotherapy because of the high rate of relapse and poor recovery, the same is not true for all patients with MOGAD. Approximately 50% of patients with MOGAD will be monophasic and recovery from relapses is superior than AQP4-Ab positive relapses. Thus, long-term immunotherapy for MOGAD is typically reserved for patients with relapsing disease or in patients with significant disability from a prior relapse (17, 60). Maintenance infusions of intravenous immunoglobulins, azathioprine and rituximab are the most commonly used agents, despite the latter being less effective in MOGAD than AQP4+NMOSD. Recommendations on the use of these treatments during pregnancy reflect those of the more studied autoimmune diseases. IL-6 targeting treatments (e.g., tocilizumab) look promising in a small number of relapsing MOGAD patients not responding to other immunosuppressant drugs (69, 70). A 3 months washout period per pharmacokinetic/pharmacodynamic placental transfer and potential risks is recommended (71).

An alternative future option may be rozanolixizumab. In a phase 2 trial in myasthenia gravis, this anti-neonatal Fc receptor humanized monoclonal antibody showed clinical improvement, suggesting a potential beneficial effect in MOGAD, which shares some pathological mechanisms. However, data on pregnancy and washout period are not currently available (72).

Limited by the relative rarity of MOG-Ab positivity, the data currently available to guide treatment decisions during pregnancy derive primarily from real-world clinical experience. Whether in the acute or non-acute phase, treatment should be personalized with consideration given to the age at conception, the severity of relapses and the disease course. Similarly, recommendations regarding resuming treatments while breastfeeding should be evaluated on a case-by-case basis.

### What should we know

#### Future areas of research

MOGAD is a newly defined disease requiring further characterization and investigation. Improving our knowledge of MOGAD pathological mechanisms is of primary importance to understand how these pathways may influence pregnancy and pregnancy-related outcomes. Likewise, further elucidation of the immunological changes in pregnancy in MOGAD patients may give us clues to the immunopathogenesis of MOGAD itself.

A significant challenge in MOGAD research is the rarity of the disease. Small sample size is the most common limitation of studies aiming at better understanding this condition. Future international, multicenter studies will be a key to collecting the number of patients necessary to systematically assess the interplay between pregnancy, the postpartum period and breastfeeding with different MOGAD phenotypes. Including women in international pregnancy registries would be of additional value. Meanwhile, sharing and publishing real-word data of experiences across centers will continue to guide pregnancy-related MOGAD issues in clinical practice.

In the future, it would be useful to identify factors associated with pregnancy-related relapses in MOGAD, as has been done for AQP4+NMOSD (41). Prospective studies with large sample size are needed to evaluate if clinical features (e.g., different phenotypes at onset, number and recovery of relapses, age at conception, treatment) or Ab-characteristics (titers, timing and persistence over time) may be independent risk factors for pregnancy-related relapses. The discovery of risk factors would facilitate earlier and more comprehensive care, including planning of laboratory studies, neuroimaging, clinical evaluations, and rehabilitation programs to reduce the impact of disease burden on MOGAD mothers, especially in the postpartum period.

Finally, there is an urgent need for evidence-based guidelines for the treatment of MOGAD. Specifically in pregnancy, the key questions that need to be addressed are (i) how do we best manage clinical relapses, (ii) do we stop and when do we stop disease modifying treatments, (iii) what pregnancy related factors may influence the decision regarding continuous immunotherapy, as the postpartum period may represent a particularly high risk for relapse, and (iv) how long do we delay restarting immunotherapy in those who are breastfeeding?

## Autoimmune encephalitis

Autoimmune encephalitis (AE) includes a group of disorders, sometimes of paraneoplastic origin, characterized by subacute onset of neurological and psychiatric manifestations associated with brain inflammation. These syndromes can be associated with antibodies targeting intracellular, synaptic or neuronal cell-surface antigens, although seronegative cases can also occur. Suggestive neurological symptoms include working memory deficits, altered mental status, focal CNS findings, seizures, movement disorders, and behavior/cognitive/autonomic dysfunctions (3). The presence of specific antibodies defines the association and frequency of cancer, age, and gender ratio (3, 73). The most common form of AE is that associated with anti-NMDAR antibodies (40% of all seropositive cases), with a median onset age of 21 years and a strong (9:1) female preponderance. This predominance is more pronounced when the onset is between puberty and menopause, when the association with tumors, in particular ovarian teratoma, is also more common. Anti-GAD-associated encephalitis also effects predominantly young women (median age 26 years-old, 9:1 female to male ratio). Anti-GABAaR or anti-mGluR5 AE also affect women of childbearing age, but are less common and show a 1:1 female to male ratio (73). As a consequence, almost all studies which have analyzed the correlation between AE and pregnancy have focused on anti-NMDAR encephalitis, with rare single cases describing AE onset during pregnancy in association with other autoantibodies (e.g., anti-AMPAR) (74).

### What do we know

#### Effect of Antibodies on fertility and pregnancy

There is no available data on fertility in women during or after AE. Although the autoimmune/inflammatory process *per se* should not directly influence fertility, residual symptoms, immunotherapy, and socioeconomic factors might have an indirect effect. Disease course (i.e., monophasic vs. relapsing or chronic disease, the latter being more common in GAD rather than NMDAR-associated encephalitis) may play a significant role in decisions regarding pregnancy (73). Of note, 94% of tumors associated with anti-NMDAR encephalitis are ovarian teratomas (75) where surgical resection is recommended. Although teratoma resection is the first treatment choice, sometimes unilateral, or bilateral oophorectomy is required, directly influencing future fertility and the need for reproductive technologies (76, 77).

Maternofetal transfer of anti-NMDAR antibodies can occur during pregnancy in both symptomatic and asymptomatic women, as supported by animal models showing reversible pathogenic effects (78). However, obstetric complications are mainly related to the severity of neurological symptoms (e.g., seizures or autonomic dysfunctions), which often require admission to an intensive care unit. Data suggest that fetal exposure to maternal antibodies rarely causes neurological complications in the developing fetus or newborn (79).

The effect of antibodies on fertility and pregnancy should be analyzed in future studies addressing this specific topic.

#### Effect of pregnancy, postpartum, breastfeeding on Ab-mediated disease

There are limited case reports and case series of patients who developed anti-NMDAR encephalitis during or after pregnancy, but of those available the disease course was similar to that observed in non-pregnant women. Few cases who have relapsed during or after pregnancy or had other concomitant autoimmune conditions have been also reported (80–83).

The possible proposed triggering factors include: changes in hormonal status (in particular the effect of estrogen and progesterone on antibody production), B cell maturation, IL10 secretion, Th1/Th2 shift, and the modification of immune tolerance induced by the embryo or placenta (84). In most cases, a good clinical outcome of the newborn and mother is reported after the administration of first line (i.e., steroids, plasma exchange, intravenous immunoglobulins—IVIG)—or second line (rituximab and in one case cyclophosphamide) treatment with no obstetric complications or fetal distress (79, 85–91). These outcomes are also in line with the authors' experience (unpublished data).

Anti-NMDAR encephalitis can occur after delivery in some patients and can be misdiagnosed as postpartum psychosis. Onset is usually reported within 3 months from delivery, regardless of multiparity, and usually occurs after normal vaginal delivery. Psychiatric symptoms (psychomotor excitement, confusion, depression, anxiety, delusions, bizarre behavior, insomnia, agitation, irritability, catatonic features, and hallucinations) are usually predominant, although this clinical phenotype and the tumor association are similar to that reported in non-pregnant women (92–95). Experts recommend systematic screening of serum anti-NMDAR antibodies in patients with acute psychosis during the postpartum period (96), in particular in those with additional neurological symptoms, EEG abnormalities or MRI signs of mesial temporal lobe involvement that support the diagnosis.

A single case of a non-paraneoplastic, treatment-responsive CASPR-2-associated encephalitis presenting with postpartum psychosis has been also described (97), suggesting that other Ab-mediated AE might occur in the postpartum period and extensive screening should be performed in these conditions.

Fetal outcome is better in patients with anti-NMDAR encephalitis in mid- to late-pregnancy, when the fetal blood-brain-barrier begins to function (84, 98) preventing the transfer of IgG1/IgG3, which are able to bind to the Fc neonatal receptor of the syncytiotrophoblasts at ~14–16 weeks of gestation. Most reports described healthy infants, but isolated neurological sequel or miscarriage/abortion have also been reported (84, 99). Despite most data not supporting an increased teratogenicity rate or fetal development delay, a higher rate of preterm delivery has been reported, mainly linked with neurological symptoms displayed by the mother (73, 79). Pre-term delivery can also be planned to reduce fetal antibody exposure and improve maternal and fetal outcomes (98). Isolated cases with respiratory distress, neural tube closing defects, and reduced body weight have been described, possibly correlated with the administration of antiepileptic and sedative treatments (79, 100). Among cases with poor fetal outcomes, a symptomatic woman who developed an anti-NMDAR encephalitis relapse in the context of a new ovarian teratoma at 37-week's gestation with a fatal fetal outcome has been described. After Cesarean section, the infant displayed hypotonia, respiratory insufficiency, and seizures followed by progressive worsening of neurological function unresponsive to IVIG. Both mother and newborn showed anti-NMDAR antibodies positivity (101).

In another case, a woman developed anti-NMDAR encephalitis at 9-weeks gestational age, delivering at 34-weeks. Despite transient improvement, she died soon after delivery and the serum anti-NMDAR positive newborn displayed movement disorders, cortical dysplasia and development delay associated with seizures at 2 years follow-up (102). In addition, an asymptomatic woman with a previous history of relapsing anti-NMDAR encephalitis at 37-weeks gestational age delivered an NMDAR positive newborn with low responsiveness and respiratory insufficiency. The infant improved spontaneously and was asymptomatic with negative anti-NMDAR serology after 1 year (103). Another anti-NMDAR positive neonate with autonomic instability has been described, but he was healthy at 1 year of age (104). Although data on the effect of NMDAR antibodies is partially discordant and the outcomes in these cases could result from a combination of factors, including the side effects of sedatives and antiepileptics drugs, anti-NMDAR testing is recommended in pregnant women with an antecedent or recent history of anti-NMDAR encephalitis.

To summarize, the onset or relapse of autoimmune encephalitis during/after pregnancy is rare and usually not associated with fetal complications.

#### Treatment strategy change during pregnancy and impact on Ab-mediated disease

Although a consensus on treatment strategies for AE in pregnancy does not exist, experts agree that timely, and prompt treatment, including tumor resection and immunotherapy, is recommended in pregnant women with AE and is related to mother and fetal outcome. Among acute therapy, IVIG, corticosteroids, and plasma exchange are usually well-tolerated as first line therapies and may reduce mother and fetus circulating antibodies (105). Among additional second line therapies azathioprine and rituximab have been administered but might retain a teratogenic and premature delivery risk, although their use is supported by studies in different autoimmune conditions during pregnancy (47, 49). Therefore, the potential benefits for the mother and the risk to the fetus should be carefully considered (73, 84). Psychotropic drugs such as haloperidol, olanzapine, lithium, and lorazepam can also improve behavioral symptoms (96). Antiepileptic drugs are also recommended, although seizures are more responsive to immunotherapy.

In patients with teratoma, laparoscopic surgery is usually the treatment of choice as it is considered curative and is not associated with fetal complications. Close fetal monitoring during this operative procedure is additionally recommended. Of note, teratoma identification might be difficult in mid- to late-pregnancy, due to the obstruction of the ultrasound field by the enlarged uterus. In these cases MRI or targeted reduced-dose computed tomography scan may be necessary to identify the teratoma (98, 106). Of note, similarly to the non-pregnant cases, cerebrospinal fluid (CSF)-restricted antibodies can be detected in pregnant/postpartum women, so that testing CSF in suspected seronegative cases is strongly recommended (88, 94, 95).

To summarize, available evidence supports prompt surgical and immunotherapy treatments in patients who develop autoimmune encephalitis during/after pregnancy, considering fetus effects in the choice of second line drugs.

### What should we know

#### Future areas of research

Literature related to pregnancy and delivery in patients with AE is scarce, usually limited to case reports, and mainly to women with anti-NMDAR encephalitis. Further multicenter studies incorporating epidemiological surveys with prolonged follow-up including detailed neuropsychological assessments of the children and mothers are paramount to defining short and long-term outcomes, treatment strategies, and clarifying the immunopathogenesis of the disease. In particular, the correlation between a mother's antibody titer and obstetric or fetal complications requires clarification in order to tailor pre-conceptual planning and postpartum follow-up. In addition, the optimal timing and type of delivery should be clarified. Finally, literature should be expanded to patients with other forms of AE.

## Conclusion

Pregnancy may influence the course of Ab-mediated disorders of the CNS. And while the manner in which this influence occurs may differ in AQP4+NMOSD, MOGAD, and AE, similarities between the three diseases can also be identified.

First, while the ability of CNS specific Ab to affect fertility is still debated due to the lack of high quality studies, disease activity and course, recovery and treatments certainly indirectly affect pregnancy (73, 107).

Second, hormonal fluctuations, particularly estrogens can potentially induce inflammation and increase pregnancy complications (42). Nonetheless, data about the role of pregnancy on all the described diseases are scarce and contrasting. On one side, a possible protective role of pregnancy on AQP4+NMOSD has been hypothesized, with a trend of reduced ARR compared to the pre-partum period. On the other side, few cases with acute events have been described in MOGAD and AE, being often the first attack ever in the former, while usually associated with other concomitant autoimmune diseases in the latter disease.

By contrast, the first postpartum trimester represents a risk for women with Ab-mediated disorders of the CNS. A rebound in the ARR was reported in NMOSD, which was higher than the pre-pregnancy period in AQP4-Ab positive patients. The onset of AE encephalitis (i.e., anti-NMDAR, or anti-CASPR-2) can occur with psychiatric symptoms soon after the delivery and can be misdiagnosed as postpartum psychosis (16, 83).

In addition, timing and type of delivery are not generally affected in AQP4+NMOSD and MOGAD, while delivery can be planned in AE to reduce fetal antibodies exposure and improve maternal and fetal outcome, as cases of fetal complications have been reported.

Finally, there are currently no evidence-based guidelines for the management of Ab-mediated disease of the CNS during pregnancy. Treatment of intrapartum relapses should be recommended, particularly when women may incur accumulation of neurological disability and functional limitations. High-dose steroids are usually well-tolerated, but close clinical surveillance should be performed to identify complications (i.e., deficit in fetal organogenesis in the first trimester, hypertension, pre-eclampsia, intrauterine growth restriction in the second, and third trimesters). Data currently available on the use of intravenous immunoglobulins during pregnancy are more reassuring than plasma exchange due to the lower risks of circulatory instability (71). Tumor resection is recommended in pregnant women with AE, while immunotherapy during pregnancy should be evaluated on a case by case, as some therapies may be dangerous for the woman and fetus.

Overall, most of the evidence related to the interplay between pregnancy and these disorders is limited to case reports. Further multicenter studies should be undertaken to clarify unresolved questions and define monitoring and treatment strategies to be used in a clinical setting.

In conclusion, in women at childbearing age with an Ab-mediated disease of the CNS, pregnancy should be carefully planned, patients and fetus routinely checked intrapartum, and a postpartum care plan organized before delivery to allow comprehensive support and reduced the burden of complications on mothers and newborns.

## Author contributions

RC designed the project and wrote the manuscript. SM and CM wrote and revised the manuscript. CT supervised the project and revised the manuscript. All authors contributed to the article and approved the submitted version.
